# Efficacy of transcranial direct current stimulation combined with gait training in patients with Parkinson’s disease: Systematic review and meta-analysis

**DOI:** 10.1017/S1092852925100618

**Published:** 2025-10-09

**Authors:** Ignacio Domínguez-Pera, David Lucena-Anton, Maria-José Estebanez-Perez, Jose-Manuel Pastora-Bernal, Rocío Martín-Valero

**Affiliations:** 1 Department of Physiotherapy, Hospital Universitario de Navarra, Pamplona, Spain; 2 Department of Nursing and Physiotherapy, University of Cadiz, Cadiz, Spain; 3 Biomedical Research and Innovation Institute of Cadiz (INiBICA), Cadiz, Spain; 4 Department of Physiotherapy, Faculty of Health Sciences, University of Granada, Granada, Spain; 5 Department of Physiotherapy, Faculty of Health Sciences, University of Málaga, CTS-1071 Research Group, Malaga, Spain

**Keywords:** transcranial direct current stimulation, Parkinson’s disease, gait training, motor symptoms

## Abstract

Parkinson’s disease, the second most prevalent neurological disorder, is a multisystem neurodegenerative disease characterized by both motor and non-motor symptoms. Transcranial direct current stimulation (tDCS) is a non-invasive brain neuromodulation technique that has been shown to be effective in some neurological conditions and for some clinical outcomes. To evaluate the efficacy of tDCS combined with gait training in Parkinson’s disease, compared to placebo, absence of treatment, conventional therapy, or other therapies. A systematic review and meta-analysis were performed in accordance with the PRISMA guidelines and registered in PROSPERO CRD42024542552. The literature search was conducted in PubMed, CINAHL, SPORT Discus, Web of Science, Scopus, MEDLINE, and Academic Search Ultimate (EBSCO) databases up to May 2024, limited to trials from the last 10 years. A total of 600 articles were identified; 9 were included in the systematic review and 8 in the meta-analysis. Significant intra-group changes were observed, but in the meta-analysis, no significant differences were seen between tDCS + gait training and tDCS placebo + gait training, although variables such as motor function slightly favored the combination (MD = −0.49; 95% CI [−1.55; 0.57], I^2^ = 0%). The combination of tDCS and gait training could provide significant motor benefits in terms of gait speed, functional mobility, cadence, motor function, quality of life, 6MWT, coordination and dynamic balance, flexibility, and stretch resistance in patients with Parkinson’s disease, but not in a more effective way than the same training without stimulation.

## Introduction

Parkinson’s disease (PD) has traditionally been categorized as a pure movement disorder, caused by the degeneration of dopaminergic neurons in the mesencephalic substantia nigra. However, recent advances in its study have led to a better understanding of the disease, which is now conceptualized as a multisystem neurodegenerative disorder, manifested through a wide variety of motor (such as bradykinesia, rest tremor, rigidity, freezing of gait, posture, balance, and gait disturbances) and non-motor symptoms (such as pain, apathy, depression, cognitive impairment, sleep disorders, olfactory dysfunction, and autonomic disorders).^1^ ^,^^2^

The “American Physical Therapy Association,” referring to patients with PD, recommends physiotherapy treatment with a very high level of evidence supporting a comprehensive approach in a community setting, including moderate- to high-intensity aerobic exercise, strength development with external stimuli, and balance and gait training.^3^ Treadmill gait training shows evidence of improvement in spatiotemporal gait parameters, while Nordic walking is supported by strong evidence of improvement in motor symptoms, balance, and different gait parameters.^4^ The inclusion of varied situations or simultaneous tasks while walking—such as dual-task gait training—has shown evidence for improving gait, motor symptoms, and balance in patients with PD.^5^

Non-invasive brain stimulation (NIBS) is currently considered a non-pharmacological option that, when used correctly, can provide significant benefits safely to patients.^6^ The transcranial direct current stimulation (tDCS) modality is one of the most commonly used.^6^ A systematic review on the treatment of patients with PD through tDCS, as well as gait training used either in isolation or combined with other therapies, showed a statistically significant improvement in gait and balance compared to placebo stimulation used alone or in combination with other therapies.^7^ tDCS can be used immediately before gait training, although its ease of use and portability during training also make these 2 therapeutic strategies perfectly compatible when combined and applied simultaneously, aiming for a potential hypothetical additive effect.^7^ To the best of our knowledge, no previous studies have considered the simultaneous use of tDCS in combination with gait training.

Therefore, the aim of this systematic review and meta-analysis is to evaluate the efficacy of the combination of tDCS with gait training in different modalities (conventional physiotherapy, with visual stimuli, under dual-task conditions, or during treadmill walking) in people with PD, as well as the different parameters and stimulation conditions that influence the results obtained.

## Methods

This systematic review and meta-analysis have been reported in accordance with the guidelines established by the PRISMA (Preferred Reporting Items for Systematic Reviews and Meta-Analyses) statement^8^ (checklist is available in Supplemental Material). The protocol was registered and updated in the PROSPERO database (International Prospective Register of Systematic Reviews), code: CRD42024542552.

An electronic literature search was conducted between February and May 2024 in PubMed, CINAHL (Cumulative Index to Nursing & Allied Health Literature), SPORT Discus, Scopus, Web of Science, MEDLINE, and Academic Search Ultimate (EBSCO) databases. The database search was restricted to articles published in the last 10 years to provide an updated overview of the field. No additional filters were applied. The PubMed search was carried out using the MeSH (Medical Subject Headings) descriptors, linked together using the Boolean operators AND and OR. The search was adapted for other databases. The detailed search strategy is available as Supplemental Material.

The standards used to determine which studies to include were based on the PICOS research strategy (Population, Intervention, Comparison, Outcome, Study Design)^9^: Population: Adult patients diagnosed with PD; Intervention: Use of tDCS in combination with unassisted gait training (conventional gait physiotherapy, under dual-task condition, gait with visual stimuli, or treadmill) performed simultaneously during the same treatment session; Comparison: Placebo, absence of treatment, conventional therapy, or other therapies; Outcome: The variables to be studied are focused on the aspects influenced by the motor symptoms present in PD (motor function, balance, gait, etc.); Study Design: Randomized controlled trials (RCTs), pilot RCT, and cross-over studies.

Articles that met the following exclusion criteria were automatically excluded from the review: (i) Publication in a language other than English or Spanish, (ii) publication within the last 10 years, and (iii) studies whose measurements were not performed in the “ON” phase (under the effects of each patient’s usual medication).

Duplicate articles were detected and removed using the “Rayyan”^10^ tool. By reviewing the title and abstract of non-duplicate articles, we excluded those with a study design other than an RCT or pilot RCT, as well as those focusing on another population or treatment method. Finally, a full-text reading of all remaining articles was performed, excluding all studies that did not meet all of the established selection criteria.

The study selection process was carried out by 2 authors (I.D.P. and R.M.V.) and, in case of doubt, the authors resolved disagreements by consensus and consulting a third author (D.L.A.) when necessary.

Regarding the extraction process, the following information was extracted from the studies: authors, year of publication, study design, sample and gender distribution, number of participants in study groups, and intervention characteristics (types, frequency, and session duration).

To evaluate the methodological quality of the RCTs included in the study, the PEDro scale was used—a specific tool for assessing clinical trials of physiotherapy interventions.^11^ The PEDro scale provides a valuable source of information to support evidence-based clinical practice in experimental studies. This scale consists of several items that identify external validity (Criterion 1, not included in the final score), internal validity (Criteria 2–9), and statistical information for interpreting the results (Criteria 10 and 11) of clinical trials. Finally, studies are classified into quality categories: low (less than 4), moderate (4–5), good (6–8), or excellent (9–10).^11^

The risk of bias for each of the selected studies was calculated using the Cochrane RevMan Web tool.^12^ The software “Review Manager (RevMan)” was used to perform the meta-analysis, in order to obtain the different forest plots to compare the changes in each variable after the interventions carried out in the different groups.^12^

Two reviewers (I.D.P. and R.M.V.) assessed the methodological quality and the risk of bias of the included studies and, in case of doubt, authors resolved disagreements by consensus and consulting a third author (D.L.A.) when necessary.

Statistical analysis was performed using Review Manager software (Version 5.4.1, developed by The Cochrane Collaboration in Copenhagen, Denmark, in 2020).^12^ The effect measure used was the mean difference (MD), reporting the results alongside their 95% confidence intervals (CIs). To assess the homogeneity, the I^2^ statistic was used, indicating 0–40% potential unimportance, 30–60% moderate heterogeneity, 50–90% substantial heterogeneity, and 75–100% considerable heterogeneity.^13^ When no heterogeneity was detected, a fixed-effect model was applied, employing a random-effects model otherwise. The significance level was set at Alpha = 0.05, and the findings were visually presented using forest plots.

Meta-analysis was performed on any variable that was measured in several of the studies included in this review, using the same or very similar measurement instruments. Where necessary and possible, and when the units of measurement used were different, the necessary conversion was performed for inclusion in the quantitative synthesis.

## Results

**Figure 1.**
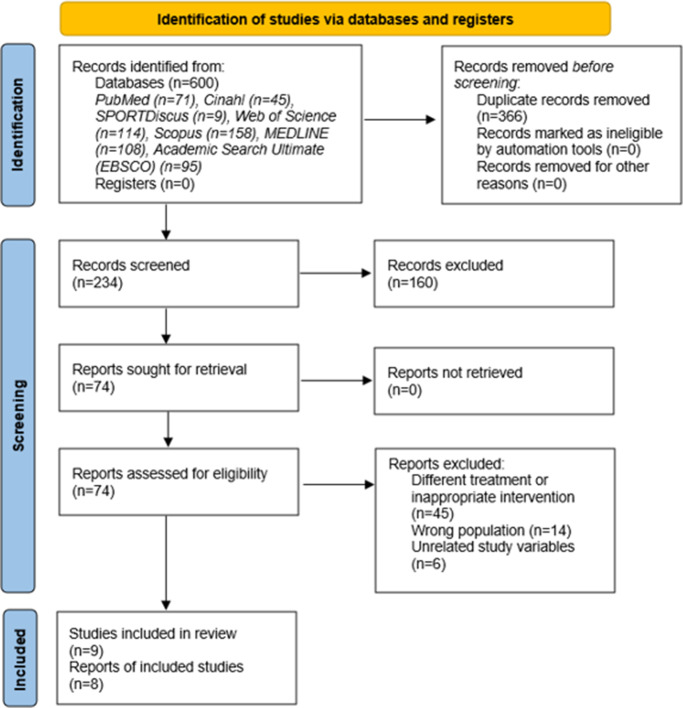
Preferred Reporting Items for Systematic Reviews and Meta-Analyses (PRISMA) flow diagram.

After searching the aforementioned databases, a total of 600 potential articles were initially obtained, which were screened according to the selection criteria until the 9 studies finally included in this systematic review were obtained. The selection of the articles included in this systematic review was based on the PRISMA recommendations, and the protocol is shown visually in the flow diagram (Figure 1).^8^

Among the 9 articles finally included in the present systematic review, 4 were RCTs,^14^ ^–^^17^ 3 P-RCTs,^18^ ^–^^20^ and 2 C-ORCTs^21^ ^,^^22^—1 of them also considered a pilot study.^22^

The total sample size across all included studies was 250 participants, with a mean of 27.78 ± 14.09 per study. The study with the largest number of participants (N = 53) is that of Yotnuengnit et al.,^16^ while the two with the smallest sample size (N = 16) were those by Schabrun et al.^19^ and Kaski et al.^22^

In all studies, the intervention differentiated a minimum of 2 groups, always randomly configured. In 5 of the studies,^14^ ^,^^17^ ^–^^20^ there was an experimental group (EG) and a control group (CG), receiving real tDCS stimulation and placebo, respectively, both in combination with the chosen gait training or physiotherapy modality. The studies published by Kaski et al.^22^ and Mishra et al.^21^ had a crossover design. In the case of Mishra et al.,^21^ one group received a tDCS session, and the other group received a tDCS placebo, in combination with treadmill gait training, with the intervention being switched between the 2 groups after a 1-week washout.

**Figure 2.**
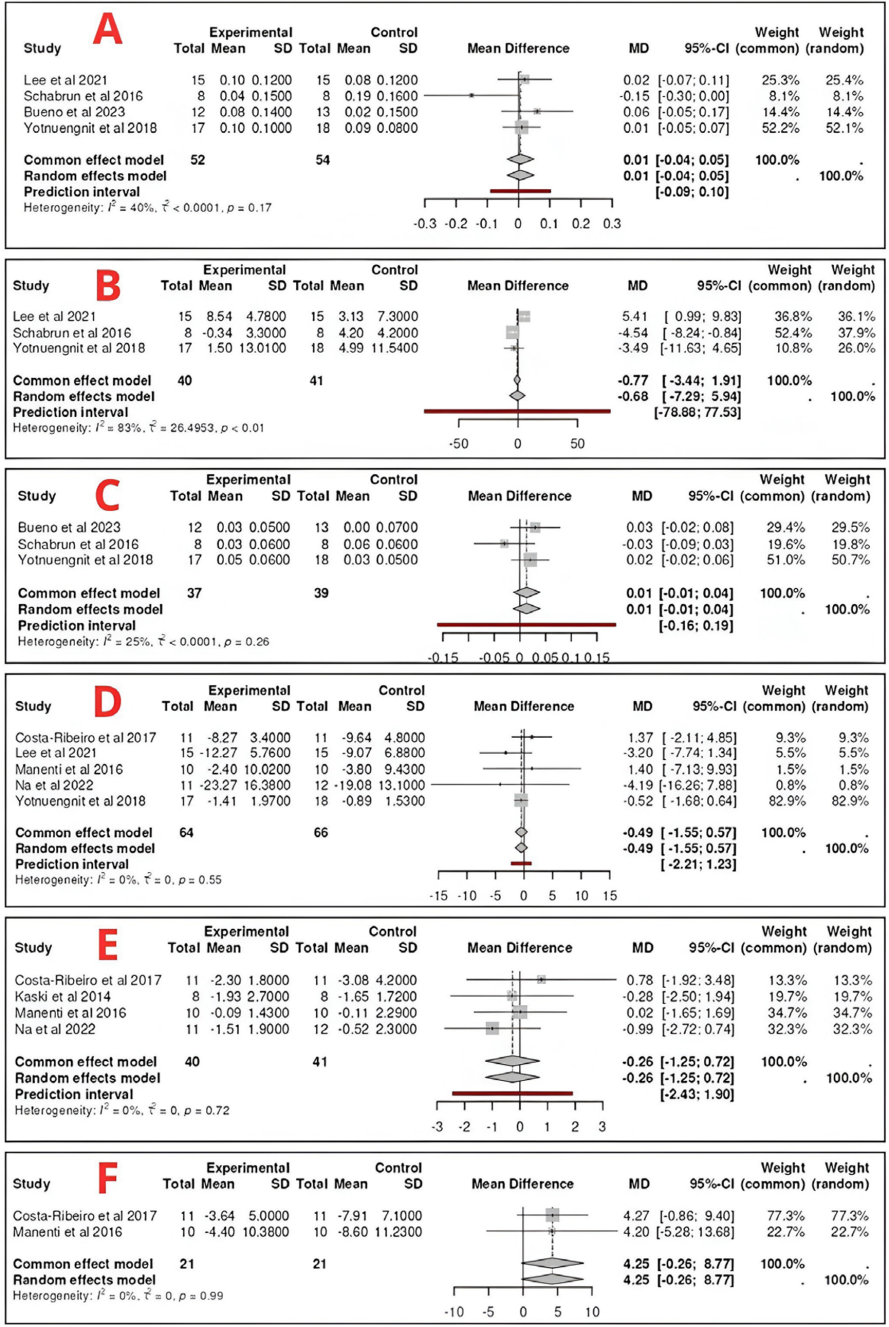
Forest plots of the variables included in the meta-analysis (A = walking speed; B = walking cadence; C = stride length; D = motor function (UPDRS III); E = functional mobility (TUGT); E = quality of life).

In the study by Kaski et al.,^22^ 1 group received physiotherapy and 1 did not, and both groups were divided into 2 subgroups, which also received either tDCS or a tDCS placebo, with the intervention being exchanged between subgroups after a 1-week washout. The remaining 2 studies had more than 2 groups, 3 in the case of Yotnuengnit et al.^16^ and 4 in the case of Bueno et al.^15^ In reference to the study by Yotnuengnit et al.,^16^ the groups included in the meta-analysis were G2 (tDCS + PT; Figure 2 as experimental) and G3 (tDCS placebo + PT, Figure 2 as control). However, in the study Bueno et al.,^15^ G1 (tDCS (Cz) + PT; Figure 2 as experimental) and G3 (tDCS placebo + PT; Figure 2 as control) were included.

The total number of sessions showed some variability between the different studies, as participants included by Bueno et al.^15^ received only 1 session, while in the study published by Lee et al.,^14^ participants received the highest number of sessions: 20. Two of the studies^14^ ^,^^20^ train gait by incorporating visual stimulation, while 2 others^19^ ^,^^21^ train gait by implementing different dual tasks. The study by Na et al.^18^ uses treadmill gait training, and the remaining 4 studies^15^ ^–^^17^ ^,^^22^ were based on physiotherapy programs, specifically incorporating gait work, in addition to other aspects amenable to physiotherapy treatment.

Table 1 details the tDCS protocol carried out in each study. In all of the studies included in the present review,^14^ ^–^^22^ a current intensity of 2 mA was used, and in all of them, the electrodes were placed according to the 10/20 EEG system guidelines.^23^ In 3 of the studies,^15^ ^,^^16^ ^,^^20^ tDCS was applied prior to gait training or physiotherapy, and in the remaining 6 studies,^14^ ^,^^17^ ^–^^19^ ^,^^21^ ^,^^22^ it was applied simultaneously while the physical intervention was being performed.

**Table 1.** Description of the Studies Included in the Systematic Review

Authors and year	Study design	Sample gender distribution	Intervention			
			Groups	tDCS	Modality PT or GT	Session/weeks session duration
Bueno et al. ^15^	RCT	N = 50 32 M–18 W	G1: tDCS (Cz) + PT (*n* = 12) G2: tDCS (C3-Cz-C4) + PT (*n* = 12) G3: tDCSP + PT (*n* = 13) G4: LE + PT (*n* = 13)	“Soterix 1x1 tDCS Low-Intensity Stimulator” (Model 1300-A, Soterix Medical, NY) G1: a-Cz, c-SO G2: a-(C3-Cz-C4), c-SO CE: 10/20 EEG system 2 mA × 20 min. tDCS before PT	PT gait and balance	A single session Follow-up after 24 h
Costa-Ribeiro et al.^20^	P-RCT	N = 22 15 M–7 W	EG: tDCS + GTVC (*n* = 11) CG: tDCSP + GTVC (*n* = 11)	“neuroConn equipment-DC”, Germany a-Cz, c-SO hf cl/ hc + affect CE: 10/20 EEG system 2 mA × 13 min tDCS before GTVC	GTVC	10 Sessions 4 Week 3 Ss/W 4-week follow-up
Kaski et al.^22^	C-ORCTP	N = 16 NS	G1: tDCS + PT (*n* = 4) tDCSP + PT (*n* = 4) Cruce G1.1 and G1.2/1 Week G2: tDCS (*n* = 4) tDCSP (*n* = 4) Crossing G2.1 and G2.2/ 1 Week	Magstim Eldith DC Stimulator (neuroConn, Ilmenau, Germany) a-Cz, r-inion CE: 10/20 EEG system 2 mA × 15 min G1: tDCS simultaneous PT	PT gait and balance	2 Ss, 1 W-WO
Lee et al.^14^	RCT	N = 30 14 M–16 W	EG: tDCS + GTVC (*n* = 15) CG: tDCSP + GTVC (*n* = 15)	DC-STIMULATOR PLUS (Neuroconn, Ilmeneau, Germany) a-Cz, c-FP2 Right Orbit CE: 10/20 EEG system 2 mA × 20 min simultaneous GTVC	GTVC	20 Ss 4 W 2-week follow-up
Mishra et al.^21^	C-ORCT	N = 20 14 M–6 W	G1: tDCS + GT (*n* = NE) G2: tDCSP + GT (*n* = NE) Cruce G1 and G2/1 Week of WO	Soterix 1×1 tDCS mini-CT Stimulator, Soterix Medical Inc., NY a-DLPFC Left, c-SO Right. CE: 10/20 EEG system 2 mA × 30 min GT pre-tDCS, simultaneous, and post-tDCS (immediate, 15, and 30 min.)	GT and dual-task	2 Ss, 1 W-WO No follow-up
Manenti et al.^17^	RCT	N = 20 11 M–9 W	EG: tDCS + PT (*n* = 10) CG: tDCSP + PT (*n* = 10)	BrainStim, EMS, Bologna, Italy a-DLPFC hf cl al hc + affect, r-SO cl CE: 10/20 EEG system 2 mA × 25 min tDCS simultaneous PT	PT gait and balance, initiation of movement, control of falls, and rhythmic movements	10 Ss 2 W 5 Ss/W 2-week and 3-month follow-ups
Na et al.^18^	RCTP	N = 23 10 M–13 W	EG: tDCS + TGT (*n* = 11) CG: tDCSP + TGT (*n* = 12)	YDS–401B, Ybrain Inc., Korea a-Cz, c-(Fz-C5-C6-Pz) CE: 10/20 EEG system 2 mA × 20 min tDCS simultaneous TGT	TGT	10 Ss 4 W 3 Ss/W 4-week follow-up
Schabrun et al.^19^	RCTP	N = 16 10 M–6 W	EG: tDCS + GT (*n* = 8) CG: tDCSP + GT (*n* = 8)	“Magtism, Uk” a-M1 Left, c-SO Right CE: 10/20 EEG system 2 mA × 20 min tDCS simultaneous GT	Gain with dual-tasks	9 Ss 3 W 3 Ss/W 12-week follow-up
Yotnuengnit et al.^16^	RCT	N = 53 33 M–20 W	G1: tDCS (*n* = 18) G2: tDCS + PT (*n* = 17) G3: tDCSP + PT (*n* = 18)	“Magstim eldith DC stimulator plus” (Magtism Company Ltd, England) a-Cz, c-SO CE: 10/20 EEG system 2 mA × 30 min tDCS followed by PT	PT gait and balance, mobility, and strengthening	6 Ss 2 W 3 Ss/W follow-up at 2 and 6 weeks

Abbreviations: RCT = randomized clinical trial; P-RCT = randomized pilot clinical trial; C-ORCT = randomized crossover clinical trial; N = sample; *n* = number of participants; M = Men; W = Women; NS = not specified; G1 = Group 1; G2 = Group 2; G3 = Group 3; G4 = Group 4; tDCS = transcranial direct current stimulation; tDCSP = transcranial placebo stimulation; PT = Physiotherapy; GE = Experimental group; CG = Control group; GT = Gait training; GTVC = Gait training with visual cues; WO=Washout; CE = Electrode placement; TGT = Treadmill gait training; Ss = Session; W = Week; Min = minute; a = anode; c = cathode; mA = milliampere; SO = supraorbital; DLPFC = dorsolateral prefrontal cortex; r = reference; hf = hemisphere; hc = hemibody; affect = affected; cl = contralateral

Application time per session ranged between 13^20^ and 30 minutes^16^ ^,^^21^ and the brain area stimulated in more studies was Cz,^14^ ^–^^16^ ^,^^18^ ^,^^20^ ^,^^22^ followed by DLPFC^17^ ^,^^21^ and M1^19^. The stimulator models used in the included studies were really variable: “Soterix 1x1 tDCS Low-Intensity Stimulator” (Model 1300-A, Soterix Medical, NY),^15^ Magstim Eldith DC Stimulator (neuroConn, Ilmenau, Germany),^22^ “YDS-401B,” Ybrain Inc, Korea,^18^ and so forth. All details related to stimulation are specified in Table 1, together with the outcome measures.

In relation to the study outcome measures, it is essential to point out that in all the articles included in this systematic review,^14^ ^–^^22^ the measurements were taken during the “ON” phase, under the effects of each patient’s usual medication. With the exception of the article by Manenti et al.,^17^ all the studies^14^ ^–^^16^ ^,^^18^ ^–^^22^ took gait speed as one of the variables to be studied, using tools such as the GAITRite System,^14^ ^,^^19^ ^,^^21^ 10MWT,^18^ ^,^^20^ Optitrack System,^15^ Qualysis Track Manager,^19^ or video analysis. Motor function was assessed in 5 of the studies^14^ ^,^^16^ ^–^^18^ ^,^^20^ using section III of the UPDRS, and 5 studies^17^ ^–^^20^ ^,^^22^ assessed functional mobility using the TUGT.

In addition, 6 of the studies^14^ ^,^^16^ ^–^^20^ in this review performed minimal follow-up assessment of outcomes between 2 and 12 weeks post-intervention, while the study by Bueno et al.^15^ followed up outcomes 24 hours after the intervention.

### Results based on meta-analysis

#### Gait speed

A meta-analysis was performed for gait speed, measured using similar tools such as GAITRITE System,^14^ ^,^^19^ Optitrack System,^15^ and Qualysis Track Manager.^16^ A p-value = 0.17 was obtained, which implies that the results are considered not statistically significant (MD = 0.01, 95% CI [−0.04; 0.05]), as well as an I^2^ value = 40%, showing a moderate degree of heterogeneity (Figure 2A). Based on the preliminary results and looking at the forest plot, no conclusive results can be established.

Individually, none of the studies^14^ ^–^^16^ ^,^^19^ showed a statistically significant difference between EG and CG. However, despite not reaching statistical significance, the forest diagram shows that in the study by Schabrun et al.,^19^ the CG obtained better results than the EG, while the opposite occurred in the trial by Bueno et al.,^15^ with the EG showing better results.

In reference to the study by Yotnuengnit et al.,^16^ the groups included in the meta-analysis were G2 (shown in Figure 2 as experimental) and G3 (shown in Figure 2 as control), as they were the most interesting for the present review, combining both the physiotherapy intervention with tDCS, real and placebo, respectively. With regard to the other study with more than 2 groups, the study by Bueno et al.^15^ decided to include groups 1 and 3 in the meta-analysis, including both physiotherapy work and placebo stimulation in the case of G3 (Figure 2 as control) or real stimulation in the case of G1 (Figure 2 as experimental), selecting this group and not G2 because it exclusively stimulates the cerebral cortex area of Cz, as in other studies included in the meta-analysis of this variable.^14^ ^,^^16^

For the study by Lee et al.,^14^ the post-intervention–pre-intervention mean difference and the corresponding standard deviation were calculated, as these data were not provided. In addition, prior to the meta-analysis, the units were converted from cm/s to m/s for the study by Lee et al.^14^

#### Gait cadence

A meta-analysis was performed for gait cadence in studies using similar tools such as the GAITRITE System^14^ ^,^^19^ and Qualysis Track Manager,^16^ with the result being inconclusive, based on the forest plot. A p-value <0.01 was obtained (MD = −0.68, 95% CI [−7.29;5.94]), as well as a value of I2 = 83%, showing a very high degree of heterogeneity (Figure 2B).

Individually, the study by Lee et al.^14^ showed a statistically significant improvement of EG over CG, while the work by Schabrun et al.^19^ showed a statistically significant difference in favor of CG.

With reference to the study by Yotnuengnit et al.,^16^ the groups included in the meta-analysis were G2 (Figure 2 as experimental) and G3 (Figure 2 as control), as they were the most interesting for the present review, both combining physiotherapy intervention with tDCS, real and placebo, respectively.

For the study by Lee et al.,^14^ the post-intervention–pre-intervention mean difference and the corresponding standard deviation were calculated, as these data were not provided.

By performing an arithmetic mean between three 60-second walks, Costa Ribeiro et al.^20^ could not find any difference between the GE and the CG, although both improved significantly after their respective interventions.

#### Step length

A meta-analysis was performed for step length in studies using similar systems, such as Optitrack System,^15^ GAITRite System,^19^ and Qualysis Track Manager.^16^ A p-value = 0.26 was obtained, which implies that the results are considered not statistically significant (MD = 0.01 m, 95% CI [−0.01;0.04]), as well as an I^2^ value = 25%, showing a moderate degree of heterogeneity (Figure 2C). Based on the preliminary results and looking at the forest plot, no conclusive results can be established.

Individually, none of the studies^15^ ^,^^16^ ^,^^19^ showed a statistically significant difference between EG and CG, with EG obtaining better results in the studies by Bueno et al.^15^ and Yotnuengnit et al.,^16^ with the CG having the greatest benefits in the article by Schabrun et al.^19^

With reference to the study by Yotnuengnit et al.^16^ the groups included in the meta-analysis were G2 (Figure 2 as experimental) and G3 (Figure 2 as control), as they were the most interesting for the present review, combining both physiotherapy intervention with tDCS, real and placebo, respectively. Regarding the other study with more than 2 groups, from the study by Bueno et al.^15^ it was decided to include groups 1 and 3 in the meta-analysis, including both physiotherapy work and placebo stimulation in the case of G3 (Figure 2 as control) or real stimulation in the case of G1 (Figure 2 as experimental), selecting this group and not G2 because it exclusively stimulates the cerebral cortex area of Cz, as in another of the studies included in the meta-analysis of this variable.^16^ In addition, prior to the meta-analysis, the conversion of units from cm to m was carried out for the study by Yotnuengnit et al.^16^

#### Motor function (UPDRS III)

A meta-analysis was performed for the data obtained through UPDRS-III.^14^ ^,^^16^ ^–^^18^ ^,^^20^ A p-value = 0.55 was obtained, which implies that the results are considered not statistically significant (MD = −0.49, 95% CI [−1.55;0.57]), as well as a value of I2 = 0%, therefore there is maximum homogeneity (Figure 2D). Based on the preliminary results and, looking at the forest diagram, no conclusive results can be established.

Individually, in none of the studies^14^ ^,^^16^ ^–^^18^ ^,^^20^ was there a statistically significant difference between EG and CG. However, in the studies by Lee et al.^14^ and Na et al.,^18^ GE showed better results, with a superior decrease in the UPDRS domain III score. Despite not showing statistically significant difference between groups (SSDBG) in the meta-analysis, Lee et al.^14^ did report it in their study being favorable to the EG.

In reference to the study by Yotnuengnit et al.,^16^ the groups included in the meta-analysis were G2 (Figure 2 as experimental) and G3 (Figure 2 as control), as they were the most interesting for the present review, combining both physiotherapy intervention with tDCS, real and placebo, respectively.

For the publications by Lee et al.^14^ and Manenti et al.,^17^ post-intervention–pre-intervention mean differences and their corresponding standard deviations were calculated, as these data were not provided.

#### Functional mobility (TUGT)

A meta-analysis was performed for the data obtained through the Timed Up and Go Test (TUGT).^17^ ^,^^18^ ^,^^20^ ^,^^22^ A p-value = 0.72 was obtained, which implies that the results are considered not statistically significant (MD = −0.26, 95% CI [−1.25;0.72]), as well as an I^2^ value = 0%, showing a maximum degree of homogeneity between studies (Figure 2E). Based on the preliminary results and looking at the forest plot, no conclusive results can be established.

Individually, none of the studies^17^ ^,^^18^ ^,^^20^ ^,^^22^ managed to show a statistically significant difference between EG and CG. However, in the study by Na et al.,^18^ the results were more positive in the EG, while in the study by Costa-Ribeiro et al.,^20^ it was the CG that obtained better results.

For the study by Manenti et al.,^17^ post-intervention–pre-intervention mean differences and their corresponding standard deviations were calculated, as these data were not provided.

The study by Kaski et al.^22^ was included in the meta-analysis, with only Group 1 being analyzed, as it was a crossover trial in which this group participated in the training session with real tDCS (figure as experimental) or placebo (figure as control), changing the type of stimulation in the following session, after a week of washout.

In the study by Schabrun et al.,^19^ no significant intra- or inter-group changes were observed.

#### Quality of life

A meta-analysis was performed for data collected through the Parkinson’s Disease Questionnaire-39 (PDQ-39).^17^ ^,^^20^ A p-value = 0.99 was obtained, which implies that the results are considered not statistically significant (MD = 4.25, 95% CI [−0.26;8.77]), as well as an I^2^ value = 0%, showing a maximum degree of homogeneity (Figure 2F). Based on the preliminary results and observing the forest plot, no conclusive results can be established; the results being favorable to EG, but not reaching the values of statistical significance.

In none of the included studies,^17^ ^,^^20^ statistically significant differences were found between the EG and CG, although the results in both studies were clearly favorable to the EG.

For inclusion in the meta-analysis of the study by Manenti et al.,^17^ post-intervention–pre-intervention mean differences and their corresponding standard deviations were calculated, as these data were not provided.

### Results based on a systematic review

#### Dual-task gait speed

Gait speed under dual-task conditions was assessed in 3 of the studies included in the review,^15^ ^,^^19^ ^,^^21^ although the conditions were highly variable, so it was decided not to include this parameter in the meta-analysis, and the results were summarized qualitatively.

Mishra et al.^21^ reported a statistically significant difference in favor of the group that received real tDCS in combination with gait work. Schabrun et al.^19^ reported significant changes in the control and experimental groups, although with no notable differences between the two. In the study by Bueno et al.^15^ no significant changes were observed in either group.

#### 6MWT

The 6MWT test was only used by Kaski et al.^22^ and showed statistically significant differences in favor of individuals who underwent the combination of tDCS with physiotherapy, compared to those who received tDCS alone or physiotherapy alone.

#### Dynamic gait index

Used to assess gait, balance, and risk of falls. The study by Na et al.^18^ failed to demonstrate a significant difference between EG and CG, although statistically significant changes were seen in CG.

#### Coordination and dynamic balance

Manenti et al found significant changes in the Four Square Step Test (FSST) after intervention and at 3-month follow-up in EG and CG, with no differences between the two reaching significance.^17^

#### Flexibility and resistance to stretching

The Sit and Reach Test (SRT) was used to show significant changes after the intervention in the CG and EG of the study by Manenti et al., with no significant differences between them.^17^

#### Risk of falls

The Functional Reach Test was used in the study by Na et al.^18^ to assess the risk of falls, testing stability, and balance dynamically, showing significant improvements in the CG, but no significant differences with respect to the experimental study.

### Risk of bias and methodological quality

**Figure 3.**
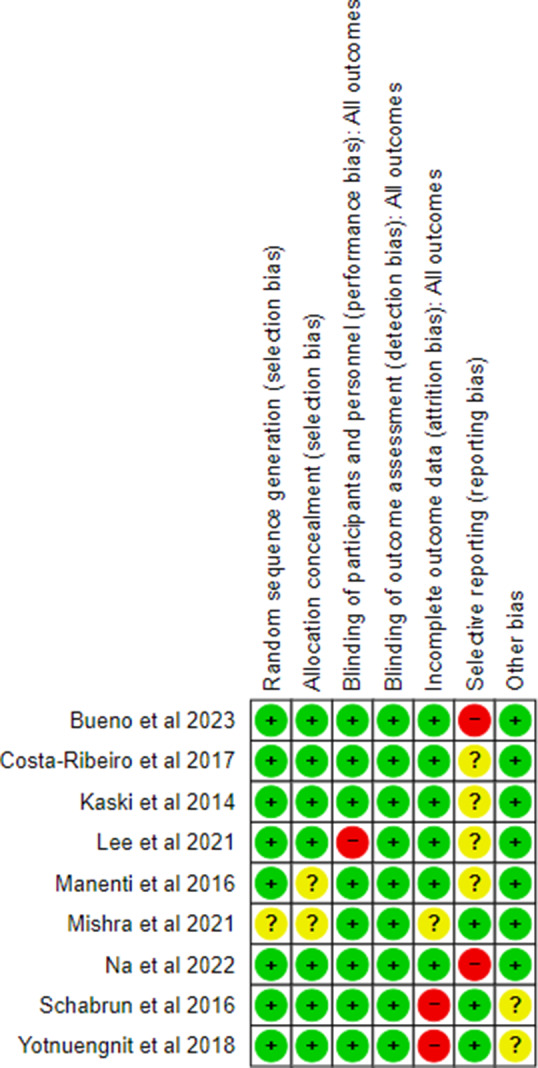
Risk of bias of the different articles included in the present systematic review and meta-analysis.

**Figure 4.**
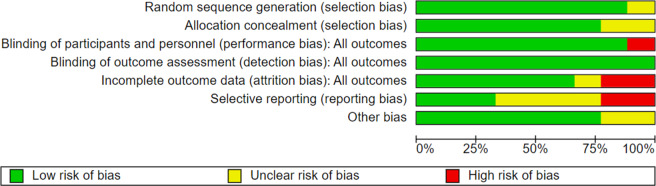
Summary and percentages of the overall risk of bias.

A risk of bias analysis was performed for each of the articles included in this review. Figure 3 details the risk of bias in the different domains for each trial. In addition, a summary of the overall risk of bias of this systematic review is presented (Figure 4).

The paper with the highest risk of bias is Mishra et al.,^21^ followed by the trials by Yotnuengnit et al.,^16^ Schabrun et al.,^19^ Manenti et al.,^17^ and Lee et al.^14^ Overall, the biases with the highest risk of occurrence were reporting bias and attrition bias, while there was a very low risk of detection bias and selection bias.

In terms of methodological quality, the mean score out of a maximum of 10 points for the studies included in this systematic review was 8.56 ± 1.51, indicating very good methodological quality. Seven of the studies included in the review^15^ ^,^^17^ ^–^^22^ presented excellent methodological quality, 2 of them^17^ ^,^^18^ obtaining the maximum score. The authors achieved the least success with Item 6, which was related to the blinding of therapy administrators. Detailed information on the score obtained by each study is shown in Table 2.

**Table 2.** PEDRO Scale Scores and Methodological Quality of the Studies Included in the Systematic Review

Study	Pedro scale criteria											Total score and methodological quality
	1	2	3	4	5	6	7	8	9	10	11	
Bueno et al.^15^	YES	YES	YES	YES	YES	NO	YES	YES	YES	YES	YES	9/10 Excellent
Costa-Ribeiro et al.^20^	YES	YES	YES	YES	YES	YES	YES	YES	NO	YES	YES	9/10 Excellent
Kaski et al.^22^	YES	YES	YES	YES	YES	NO	YES	YES	YES	YES	YES	9/10 Excellent
Lee et al.^14^	YES	YES	NO	YES	YES	NO	NO	YES	NO	YES	YES	6/10 Good
Mishra et al.^21^	YES	YES	NO	YES	YES	YES	YES	YES	YES	YES	YES	9/10 Excellent
Manenti et al.^17^	YES	YES	YES	YES	YES	YES	YES	YES	YES	YES	YES	10/10 Excellent
Na et al.^18^	YES	YES	YES	YES	YES	YES	YES	YES	YES	YES	YES	10/10 Excellent
Schabrun et al. ^19^	YES	YES	YES	NO	YES	YES	YES	YES	YES	YES	YES	9/10 Excellent
Yotnuengnit et al.^16^	YES	YES	NO	YES	NO	NO	YES	YES	NO	YES	YES	6/10 Good

1. Choice criteria were specified. 2. Subjects were randomly assigned to groups (in a crossover study, subjects were randomly distributed as they received treatments). 3. Allocation was concealed. Groups were similar at baseline for the most important prognostic indicators. 5. All subjects were blinded. 6. All therapists administering therapy were blinded. 7. All assessors measuring at least 1 key outcome were blinded. 8. Measures of at least 1 of the key outcomes were obtained from more than 85% of the subjects initially assigned to the groups. 9. Results were presented for all subjects who received treatment or were assigned to the control group, or when this could not be done, data for at least 1 key outcome were analyzed on an “intention-to-treat” basis. 10. Results of statistical comparisons between groups were reported for at least 1 key outcome. 11. The study provides point measures and measures of variability for at least 1 key outcome.

## Discussion

This systematic review and meta-analysis have been developed with the aim of finding out the effectiveness of combining tDCS with gait training in patients with PD, analyzing a total of 12 variables; 6 of which were finally included in the meta-analysis. To our knowledge, the present study is the first systematic review and meta-analysis to analyze the effectiveness of the simultaneous combination of both techniques performed during the treatment session in patients with PD.

A total of 9 studies were analyzed in systematic reviews, of which 8 provided quantitative data for the meta-analysis. Although no statistically significant differences between groups were found for any of the study variables, slightly favorable results were obtained for the combination of tDCS and gait work in terms of step length and motor function (UPDRS III), although certain results were also more favorable to the control group, namely in quality of life (PDQ-39).

Based on the qualitative synthesis, a statistically significant and favorable difference was observed for the combination of tDCS and gait training in selected items in terms of gait speed under dual-task conditions,^21^ dynamic gait balance (FGA),^14^ and 6MWT^22^ compared to gait work with placebo stimulation. Likewise, the variables showing intra-group clinical significance were very similar in CG and EG, and the same was concluded for the follow-up assessments.

In order to contextualize and compare the results observed in the present study, a comparison was made with those obtained by other systematic reviews and meta-analyses, which shared the same intervention and/or population.

Coinciding with the results obtained in 2021 by the review of Rodrigues et al.,^24^ after performing the present MA, it cannot be established that the addition of tDCS to gait training provides greater gains in terms of speed to patients with PD than the same training performed in isolation. This was replicated in terms of step length and cadence.

There is currently no well-established protocol for tDCS in PD, and it is essential to consider differences in terms of stimulation when interpreting the results obtained.

In relation to physiotherapy intervention, although gait work was the epicenter of all the protocols, the selected studies used different alternatives for combination with tDCS:

### Gait training with visual stimuli

The 2 studies^14^ ^,^^20^ also coincided in the areas of stimulation (a-Cz; c-SO), which facilitates the comparison and interpretation of the results. It has been observed that the time invested is the same, 4 weeks, while the duration of the session was 13 minutes in one,^20^ and 20^14^ in the other. Walking speed was assessed by the 2 studies using very different measuring instruments (10MWT^20^ and GAITRite System^14^), making comparison difficult. In terms of cadence, both studies observed clinical significance after the EG intervention, although only Lee et al.^14^ found a SSDBG in favor of EG (F = 5.944; p = 0.021).

Based on the comparison of these two articles, and although the samples are small, we can suggest a greater efficacy of the combination of gait training with visual stimuli and tDCS when applied simultaneously (coinciding with the observations of Mitsutake et al.^25^), at a rate of 5 sessions per week and with an approximate duration of each stimulation session of 20 minutes.

### Gait training with dual tasks

Mishra et al.^21^ and Schabrun et al.^19^ implemented various dual-task contexts in gait training, although the design of each study was very different, making it difficult to draw conclusions. The only variable studied in the study by Mishra et al.^21^ was gait speed, in normal conditions and under dual task, using the GAITRite System for its assessment, coinciding with the tool used by Schabrun et al.^19^ Under normal conditions, Mishra et al.^21^ reported clinical significance in walking speed in both groups, with no SSDBG, while under dual-task condition, both studies reported clinical significance in speed, although a SSDBG was only observed in the study by Mishra et al.^21^ (p = 0.017 after 15 min; p < 0.01 after 30 min).

### Treadmill gait training

The only study to incorporate treadmill gait training was that of Na et al.,^18^ applied simultaneously with tDCS. A total of 10 sessions were carried out over a month, coinciding with Costa-Ribeiro et al.,^20^ with both also having the anode in Cz, although they differed in the arrangement of the cathodes. Both studies assessed functional mobility through TUGT, with significant changes appearing in the EGs of both studies, without PEGD. In relation to motor function (UPDRS III), in both studies, clinical significance was obtained in EG and CG, without SSDBG.

Finally, the remaining four studies^15^ ^–^^17^ ^,^^22^ performed a physiotherapy protocol focused on gait work and the aspects necessary for gait improvement. The application of tDCS and the duration and frequency of treatment showed great variability between the 4 trials, making it difficult to compare them.

Based on the above, despite the heterogeneity of the different studies, certain recommendations or suggestions can be established for clinical practice when combining tDCS with gait training in patients with PD. The use of a current of 2 mA is sufficient to achieve significant changes. It is recommended that electrode placement be carried out according to the indications of the “10/20 EEG” System. Conventional physiotherapy work focuses on gait training with dual tasks, visual stimuli, and treadmill stimuli. Whenever possible and safe, combine both interventions simultaneously, applying stimulation at the same time as active gait training.

### Limitations and future recommendations

This systematic review has certain limitations, the most notable being the small number of articles available and the marked heterogeneity among them, as well as the impossibility of including all the study variables in the meta-analysis, due to the variability present in the different measurement instruments or assessment conditions.

Another limitation of this review lies in the need to carry out certain operations and conversions of units of measurement to include certain studies in the quantitative synthesis, increasing the risk of the appearance of certain biases.

Based on what has been observed, it is considered necessary to carry out a new RCT with larger population samples and homogeneity in terms of tDCS application. It is essential to create a unified tDCS protocol for PD that establishes a common basis to be followed in relation to the time of application of stimulation, the minimum number of sessions, recommended frequency of these, and, especially, recommendations for the arrangement of the anode and cathode, clarifying the potential benefits to be obtained depending on the chosen location. In addition, in order to enhance the benefits to be obtained, it is considered of great interest to carry out trials comparing different types of gait training, such as those mentioned in this work, as well as other different ones, under the same conditions and guidelines for the application of tDCS.

## Conclusion

In conclusion, it can be established that the combination of tDCS with different gait training modalities can produce improvements in gait speed, gait cadence, step length, motor function, functional mobility, and quality of life in people with PD, although there was no statistically significant differences with respect to the same training carried out in a conventional manner, without combining it with tDCS. The main protocols used were gait training with visual stimuli, gait training with dual tasks, and treadmill training, obtaining benefits on gait cadence, gait speed, and motor function, respectively.

The inclusion of this combined therapy in clinical practice could have an impact on the rehabilitation of people with PD. However, based on our meta-analysis results, further research is required to provide scientific evidence on this topic.

## Supporting information

Domínguez-Pera et al. supplementary material 1 Domínguez-Pera et al. supplementary material

Domínguez-Pera et al. supplementary material 2 Domínguez-Pera et al. supplementary material

## Data Availability

Not applicable (this is a review paper).

## Abbreviation

6MWT: 6-minute walk test
10MWT: 10-meter walk test
A: anode
Affect: affected
AVD: activities of daily living
BBS: Berg balance scale
C: cathode
C-ORCT: cross-over randomized clinical trial
CI: confidence interval
Cl: contralateral
DLPFC: dorsolateral prefrontal cortex
DGI: dynamic gait index
Ec: eyes closed
EG: experimental group
Eo: eyes open
EP: electrode placement
ER: educational reading
F: F-statistic
FGA: functional gait assessment
FOG: freezing of gait
FOG-Q: freezing of gait questionnaire
FRT: functional reach test
FSST: four square step test
G1: group 1
G2: group 2
G3: group 3
G4: group 4
GC: control group
GT: gait training
GTVC: gait training with visual cues
I^2^: heterogeneity index
Hb: hemibody
Hp: hemisphere
M: men
mA: milliamperes
MA-ADL: motor aspects in activities of daily living
MD: mean difference
Ms: months
N: sample
NE: not specified
p: p-value
P-RCT: pilot Randomized Clinical Trial
P-tDCS: placebo transcranial direct current stimulation
PDQ-39: Parkinson’s disease questionnaire-39
PI: post-intervention
PT: physical therapy
r: correlation coefficient
R: reference
RCT: randomized clinical trial
SC: significant changes
SO: supraorbital
SRT: sit and reach test
Ss: sessions
SSDBG: statistically significant difference between groups
SST: standing stork test
tDCS: transcranial direct current stimulation
TGT: treadmill gait training
TUGT: timed up and go test
UL-MT: upper limb-motor task
UPDRS: unified Parkinson’s disease rating scale
W: week
W: women
WO: washout

## Supplementary material

The supplementary material for this article can be found at http://doi.org/10.1017/S1092852925100618.
