# Supplementary material for: Efficacy of transcranial direct current stimulation combined with gait training in patients with Parkinson’s disease: Systematic review and meta-analysis
Source: CNS Spectr. 2025 Oct 9;30(1):e79. doi: 10.1017/S1092852925100618 (PMC13064760; doi:10.1017/S1092852925100618)
Supplement: Domínguez-Pera et al. supplementary material 1 — Domínguez-Pera et al. supplementary material [file S1092852925100618sup001.docx]

**Supplemental Material 1.** Search strategy.

| **Database** | **Number of Articles** | **Search strategy** |
| --- | --- | --- |
| PubMed | 71 | ("Parkinson* Disease" OR "Idiopathic Parkinson* Disease" OR "Primary Parkinsonism") AND ("Transcranial Direct Current Stimulation" OR "tDCS" OR "Transcranial Random Noise Stimulation" OR "Transcranial Alternating Current Stimulation" OR "Transcranial Electrical Stimulation*" OR "Repetitive Transcranial Electrical Stimulation") AND ("Exercise" OR “Physical Activity*” OR “Physical Exercise*” OR “Acute Exercise*” OR “Aerobic Exercise*” OR “Exercise Training*” OR "Training" OR "Gait*" OR “Walking” OR “Ambulation” OR "Physical Therapy" OR “Exercise Therapy” OR “Rehabilitation Exercise*” OR “Resistance Training” OR “Strength Training”) |
| CINAHL | 45 |  |
| SPORT Discus | 9 |  |
| Web of Science | 114 |  |
| Scopus | 158 |  |
| MEDLINE | 108 |  |
| (Academic Search Ultimate (EBSCO)) | 95 |  |

## 
